# Supplementary material for: Hybrid Models and Biological Model Reduction with PyDSTool
Source: PLoS Comput Biol. 2012 Aug 9;8(8):e1002628. doi: 10.1371/journal.pcbi.1002628 (PMC3415397; doi:10.1371/journal.pcbi.1002628)
Supplement: Text S4 — Complete source code for the PyDSTool package (version 0.88.120504). Includes API documentation and help files linking to web pages. This file is identical to the current public release on Sourceforge.net. (ZIP) [file pcbi.1002628.s004.zip › PyDSTool/html/PyDSTool.Toolbox.neuralcomp-module.html]

xml version="1.0" encoding="ascii"?


PyDSTool.Toolbox.neuralcomp


| Home | Trees | Indices | Help | PyDSTool |
| --- | --- | --- | --- | --- |

Package PyDSTool ::
Package Toolbox ::
Module neuralcomp

[hide private]

[frames] | no frames]

# Module neuralcomp

source code

```
    An example set of basic compartmental model ModelSpec classes
    for use in computational neuroscience modeling.

    Basic classes provided:
    channel, compartment

    For multi-compartment models:
    soma, dendr_compartment, synapse, neuron, network, exc_synapse, inh_synapse

Rob Clewley, September 2005.
```


|  |  |
| --- | --- |
| Classes | [hide private] |

compatODEComponent

compatODELeafComponent

channel

compartment

channel\_on

channel\_off

soma

dendr\_compartment

neurite\_compartment

neuron

synapse

exc\_synapse

inh\_synapse

pnnetwork

network


|  |  |
| --- | --- |
| Functions | [hide private] |

|  |  |
| --- | --- |
| disconnectSynapse(syn, mspec)  Disconnect synapse object syn from ModelSpec object mspec in which it has been declared, and remove its target cell's synaptic channels. | source code |

|  |  |
| --- | --- |
| connectWithSynapse(synname, syntypestr, source\_cell, dest\_cell, dest\_compartment\_name=`'``'`, threshfun=None, alpha=None, beta=None, threshfun\_d=None, alpha\_d=None, beta\_d=None, adapt\_typestr=None, vrev=None, g=None, noauxs=True, subclass=<class 'PyDSTool.Toolbox.neuralcomp.channel'>)  Make a chemical or electrical synapse between two neurons. | source code |

|  |  |
| --- | --- |
| makeSynapseChannel(name, gatevarname=None, voltage=`'``V``'`, typestr=None, vrev=None, g=None, noauxs=True, subclass=<class 'PyDSTool.Toolbox.neuralcomp.channel'>, gamma1=None, gamma2=None)  Make a chemical or electrical (gap junction) synapse channel in a soma. | source code |

|  |  |
| --- | --- |
| makeExtInputCurrentChannel(name, noauxs=True, subclass=<class 'PyDSTool.Toolbox.neuralcomp.channel'>, gamma1=None, gamma2=None)  External input signal used directly as a current. | source code |

|  |  |
| --- | --- |
| makeExtInputConductanceChannel(name, voltage=`'``V``'`, g=None, vrev=None, noauxs=True, subclass=<class 'PyDSTool.Toolbox.neuralcomp.channel'>, gamma1=None, gamma2=None)  External input signal used as a conductance. | source code |

|  |  |
| --- | --- |
| makeBiasChannel(name, I=None, noauxs=True, subclass=<class 'PyDSTool.Toolbox.neuralcomp.channel'>, gamma1=None, gamma2=None)  Constant bias / applied current "channel". | source code |

|  |  |
| --- | --- |
| makeChannel\_halfact(name, voltage=`'``V``'`, s=None, isinstant=False, sinf=None, taus=None, spow=1, s2=None, isinstant2=False, sinf2=None, taus2=None, spow2=1, vrev=None, g=None, parlist=None, noauxs=True, subclass=<class 'PyDSTool.Toolbox.neuralcomp.channel'>, gamma1=None, gamma2=None)  Make an ionic membrane channel using the steady state and rate function formalism. | source code |

|  |  |
| --- | --- |
| makeChannel\_rates(name, voltage=`'``V``'`, s=None, isinstant=False, arate=None, brate=None, spow=1, s2=None, isinstant2=False, arate2=None, brate2=None, spow2=1, vrev=None, g=None, parlist=None, noauxs=True, subclass=<class 'PyDSTool.Toolbox.neuralcomp.channel'>, gamma1=None, gamma2=None)  Make an ionic membrane channel using the forward and backward rate formalism. | source code |

|  |  |
| --- | --- |
| makeSoma(name, voltage=`'``V``'`, channelList=None, C=None, noauxs=True, subclass=<class 'PyDSTool.Toolbox.neuralcomp.soma'>, channelclass=<class 'PyDSTool.Toolbox.neuralcomp.channel'>)  Build a soma type of "compartment" from a list of channels and a membrane capacitance C, using the local voltage name. | source code |

|  |  |
| --- | --- |
| makeDendrite(name, voltage=`'``V``'`, channelList=None, C=None, noauxs=True, subclass=<class 'PyDSTool.Toolbox.neuralcomp.dendr\_compartment'>, channelclass=<class 'PyDSTool.Toolbox.neuralcomp.channel'>)  Build a dendrite type of "compartment" from a list of channels and a membrane capacitance C, using the local voltage name. | source code |

|  |  |
| --- | --- |
| makeNeurite(name, voltage=`'``V``'`, channelList=None, C=None, noauxs=True, subclass=<class 'PyDSTool.Toolbox.neuralcomp.dendr\_compartment'>, channelclass=<class 'PyDSTool.Toolbox.neuralcomp.channel'>)  Build a neurite type of "compartment" from a list of channels and a membrane capacitance C, using the local voltage name. | source code |

|  |  |
| --- | --- |
| makePointNeuron(name, voltage=`'``V``'`, channelList=None, synapseList=None, C=None, noauxs=True)  Factory function for single compartment neurons ("point neurons"). | source code |

|  |  |
| --- | --- |
| makePointNeuronNetwork(name, componentList)  Factory function returning a pnnetwork type object from a list of compatible components (somas and synapses). | source code |

|  |  |
| --- | --- |
| makeNeuronNetwork(name, neuronList)  Factory function returning a network type object from a list of compatible components (neurons). | source code |

|  |  |
| --- | --- |
| makeSynapse(name, gatevar, precompartment, typestr, threshfun=None, alpha=None, beta=None, targetchannel=None, evalopt=True, noauxs=True)  Make a chemical synapse channel object. | source code |

|  |  |
| --- | --- |
| makeAdaptingSynapse(name, gatevar, adaptvar, precompartment, typestr, adapt\_typestr, threshfun=None, alpha=None, beta=None, threshfun\_d=None, alpha\_d=None, beta\_d=None, targetchannel=None, evalopt=True, noauxs=True)  Make an adapting chemical synapse channel object. | source code |

|  |  |
| --- | --- |
| makePowerSpec(v, p) | source code |

|  |  |
| --- | --- |
| makePar(parname, val=None) | source code |

|  |  |
| --- | --- |
| makeFun(funname, sig, defn) | source code |


|  |  |
| --- | --- |
| Variables | [hide private] |

compatGens = `['Radau_ODEsystem', 'ADMC_ODEsystem', 'Vode_ODEsy...`

voltage = `'V'`

V = `Var V (ExpFuncSpec)`

ALLOW\_THREADS = `1`

Abs = `Abs (ModelSpec wrapper)`

Acos = `Acos (ModelSpec wrapper)`

Asin = `Asin (ModelSpec wrapper)`

Atan = `Atan (ModelSpec wrapper)`

Atan2 = `Atan2 (ModelSpec wrapper)`

BUFSIZE = `10000`

Betavariate = `Betavariate (ModelSpec wrapper)`

CLIP = `0`

Ceil = `Ceil (ModelSpec wrapper)`

Choice = `Choice (ModelSpec wrapper)`

Cos = `Cos (ModelSpec wrapper)`

Cosh = `Cosh (ModelSpec wrapper)`

Degrees = `Degrees (ModelSpec wrapper)`

E = `QuantSpec e (ExpFuncSpec)`

ERR\_CALL = `3`

ERR\_DEFAULT = `0`

ERR\_DEFAULT2 = `2084`

ERR\_IGNORE = `0`

ERR\_LOG = `5`

ERR\_PRINT = `4`

ERR\_RAISE = `2`

ERR\_WARN = `1`

Exp = `Exp (ModelSpec wrapper)`

Expovariate = `Expovariate (ModelSpec wrapper)`

FLOATING\_POINT\_SUPPORT = `1`

FPE\_DIVIDEBYZERO = `1`

FPE\_INVALID = `8`

FPE\_OVERFLOW = `2`

FPE\_UNDERFLOW = `4`

Fabs = `Fabs (ModelSpec wrapper)`

False\_ = `False`

Floor = `Floor (ModelSpec wrapper)`

Fmod = `Fmod (ModelSpec wrapper)`

Frexp = `Frexp (ModelSpec wrapper)`

Gammavariate = `Gammavariate (ModelSpec wrapper)`

Gauss = `Gauss (ModelSpec wrapper)`

Getrandbits = `Getrandbits (ModelSpec wrapper)`

Getstate = `Getstate (ModelSpec wrapper)`

Hypot = `Hypot (ModelSpec wrapper)`

Infinity = `inf`

Jumpahead = `Jumpahead (ModelSpec wrapper)`

Ldexp = `Ldexp (ModelSpec wrapper)`

Log = `Log (ModelSpec wrapper)`

Log10 = `Log10 (ModelSpec wrapper)`

Lognormvariate = `Lognormvariate (ModelSpec wrapper)`

MAXDIMS = `32`

Max = `Max (ModelSpec wrapper)`

Min = `Min (ModelSpec wrapper)`

Modf = `Modf (ModelSpec wrapper)`

NAN = `nan`

NINF = `-inf`

NZERO = `-0.0`

Normalvariate = `Normalvariate (ModelSpec wrapper)`

PINF = `inf`

PZERO = `0.0`

Paretovariate = `Paretovariate (ModelSpec wrapper)`

Pi = `QuantSpec pi (ExpFuncSpec)`

Pow = `Pow (ModelSpec wrapper)`

RAISE = `2`

Radians = `Radians (ModelSpec wrapper)`

Randint = `Randint (ModelSpec wrapper)`

Random = `Random (ModelSpec wrapper)`

Randrange = `Randrange (ModelSpec wrapper)`

SHIFT\_DIVIDEBYZERO = `0`

SHIFT\_INVALID = `9`

SHIFT\_OVERFLOW = `3`

SHIFT\_UNDERFLOW = `6`

Sample = `Sample (ModelSpec wrapper)`

ScalarType = `(<type 'int'>, <type 'float'>, <type 'complex'>, ...`

Seed = `Seed (ModelSpec wrapper)`

Setstate = `Setstate (ModelSpec wrapper)`

Shuffle = `Shuffle (ModelSpec wrapper)`

Sin = `Sin (ModelSpec wrapper)`

Sinh = `Sinh (ModelSpec wrapper)`

Sqrt = `Sqrt (ModelSpec wrapper)`

Sum = `Sum (ModelSpec wrapper)`

Systemrandom = `Systemrandom (ModelSpec wrapper)`

Tan = `Tan (ModelSpec wrapper)`

Tanh = `Tanh (ModelSpec wrapper)`

True\_ = `True`

UFUNC\_BUFSIZE\_DEFAULT = `10000`

UFUNC\_PYVALS\_NAME = `'UFUNC_PYVALS'`

Uniform = `Uniform (ModelSpec wrapper)`

Vonmisesvariate = `Vonmisesvariate (ModelSpec wrapper)`

WRAP = `1`

Weibullvariate = `Weibullvariate (ModelSpec wrapper)`

Wichmannhill = `Wichmannhill (ModelSpec wrapper)`

absolute = `<ufunc 'absolute'>`

add = `<ufunc 'add'>`

bitwise\_and = `<ufunc 'bitwise_and'>`

bitwise\_not = `<ufunc 'invert'>`

bitwise\_or = `<ufunc 'bitwise_or'>`

bitwise\_xor = `<ufunc 'bitwise_xor'>`

c\_ = `<numpy.lib.index_tricks.CClass object at 0x1154330>`

cast = `{<type 'numpy.int64'>: <function <lambda> at 0x10a8bb0>...`

conj = `<ufunc 'conjugate'>`

conjugate = `<ufunc 'conjugate'>`

copysign = `<ufunc 'copysign'>`

deg2rad = `<ufunc 'deg2rad'>`

divide = `<ufunc 'divide'>`

equal = `<ufunc 'equal'>`

exp2 = `<ufunc 'exp2'>`

expm1 = `<ufunc 'expm1'>`

floor\_divide = `<ufunc 'floor_divide'>`

fmax = `<ufunc 'fmax'>`

fmin = `<ufunc 'fmin'>`

greater\_equal = `<ufunc 'greater_equal'>`

index\_exp = `<numpy.lib.index_tricks.IndexExpression object at ...`

inf = `inf`

infty = `inf`

invert = `<ufunc 'invert'>`

isinf = `<ufunc 'isinf'>`

left\_shift = `<ufunc 'left_shift'>`

little\_endian = `True`

log1p = `<ufunc 'log1p'>`

logaddexp = `<ufunc 'logaddexp'>`

logaddexp2 = `<ufunc 'logaddexp2'>`

logical\_and = `<ufunc 'logical_and'>`

logical\_not = `<ufunc 'logical_not'>`

logical\_xor = `<ufunc 'logical_xor'>`

maximum = `<ufunc 'maximum'>`

mgrid = `<numpy.lib.index_tricks.nd_grid object at 0x113d490>`

minimum = `<ufunc 'minimum'>`

multiply = `<ufunc 'multiply'>`

n = `9`

nan = `nan`

nbytes = `{<type 'numpy.int64'>: 8, <type 'numpy.int16'>: 2, <t...`

negative = `<ufunc 'negative'>`

newaxis = `None`

nextafter = `<ufunc 'nextafter'>`

not\_equal = `<ufunc 'not_equal'>`

ogrid = `<numpy.lib.index_tricks.nd_grid object at 0x113dbb0>`

ones\_like = `<ufunc 'ones_like'>`

r\_ = `<numpy.lib.index_tricks.RClass object at 0x11542f0>`

rad2deg = `<ufunc 'rad2deg'>`

reciprocal = `<ufunc 'reciprocal'>`

remainder = `<ufunc 'remainder'>`

right\_shift = `<ufunc 'right_shift'>`

rint = `<ufunc 'rint'>`

s\_ = `<numpy.lib.index_tricks.IndexExpression object at 0x11543f0>`

sctypeDict = `{0: <type 'numpy.bool_'>, 1: <type 'numpy.int8'>,...`

sctypeNA = `{'?': 'Bool', 'B': 'UInt8', 'Bool': <type 'numpy.bo...`

sctypes = `{'complex': [<type 'numpy.complex64'>, <type 'numpy....`

signbit = `<ufunc 'signbit'>`

spacing = `<ufunc 'spacing'>`

square = `<ufunc 'square'>`

subtract = `<ufunc 'subtract'>`

t = `'0'`

true\_divide = `<ufunc 'true_divide'>`

trunc = `<ufunc 'trunc'>`

typeDict = `{0: <type 'numpy.bool_'>, 1: <type 'numpy.int8'>, 2...`

typeNA = `{'?': 'Bool', 'B': 'UInt8', 'Bool': <type 'numpy.bool...`

typecodes = `{'All': '?bhilqpBHILQPfdgFDGSUVOMm', 'AllFloat': '...`


|  |  |
| --- | --- |
| Function Details | [hide private] |

|  |  |
| --- | --- |
| connectWithSynapse(synname, syntypestr, source\_cell, dest\_cell, dest\_compartment\_name=`'``'`, threshfun=None, alpha=None, beta=None, threshfun\_d=None, alpha\_d=None, beta\_d=None, adapt\_typestr=None, vrev=None, g=None, noauxs=True, subclass=<class 'PyDSTool.Toolbox.neuralcomp.channel'>) | source code |

```
Make a chemical or electrical synapse between two neurons. For
gap junctions this function returns None, otherwise will return the
synapse gating variable object.

Valid source_cell and dest_cell is a neuron or, for point neurons only,
  a soma is allowed.
The optional argument dest_compartment_name is a declared name in
dest_cell, or by default will be the soma.

The standard assumed rate equation for a chemical synapse conductance is

   s' = (1-alpha) * s - beta * s

Use the _d versions of threshfun, alpha, and beta, for an adapting chemical
synapse type's adapting variable.
```

|  |  |
| --- | --- |
| makeSynapseChannel(name, gatevarname=None, voltage=`'``V``'`, typestr=None, vrev=None, g=None, noauxs=True, subclass=<class 'PyDSTool.Toolbox.neuralcomp.channel'>, gamma1=None, gamma2=None) | source code |

Make a chemical or electrical (gap junction) synapse channel in a
soma. To select these, the typestr argument must be one of 'exc', 'inh',
or 'gap'. For a user-defined chemical synapse use a different string name
for typestr. For gap junctions, use the pre-synaptic membrane potential's
soma ModelSpec object or its full hierarchical name string for the
argument vrev.

Returns a channel-type object by default, or some user-defined
subclass if desired.

|  |  |
| --- | --- |
| makeExtInputCurrentChannel(name, noauxs=True, subclass=<class 'PyDSTool.Toolbox.neuralcomp.channel'>, gamma1=None, gamma2=None) | source code |

External input signal used directly as a current. Supply the Input
having coordinate name 'ext\_input'.

Returns a channel-type object by default, or some user-defined
subclass if desired.

|  |  |
| --- | --- |
| makeExtInputConductanceChannel(name, voltage=`'``V``'`, g=None, vrev=None, noauxs=True, subclass=<class 'PyDSTool.Toolbox.neuralcomp.channel'>, gamma1=None, gamma2=None) | source code |

External input signal used as a conductance. Supply the Input having
coordinate name 'ext\_input'.

Returns a channel-type object by default, or some user-defined
subclass if desired.

|  |  |
| --- | --- |
| makeBiasChannel(name, I=None, noauxs=True, subclass=<class 'PyDSTool.Toolbox.neuralcomp.channel'>, gamma1=None, gamma2=None) | source code |

Constant bias / applied current "channel".

Returns a channel-type object by default, or some user-defined
subclass if desired.

|  |  |
| --- | --- |
| makeChannel\_halfact(name, voltage=`'``V``'`, s=None, isinstant=False, sinf=None, taus=None, spow=1, s2=None, isinstant2=False, sinf2=None, taus2=None, spow2=1, vrev=None, g=None, parlist=None, noauxs=True, subclass=<class 'PyDSTool.Toolbox.neuralcomp.channel'>, gamma1=None, gamma2=None) | source code |

```
Make an ionic membrane channel using the steady state and rate function formalism.

i.e., that the gating variable s has a differential equation in the form:
   s' = (sinf(v) - v)/taus(v)
The channel may have up to two gating variables, each of which is given by an ODE.

If either gating variable has its corresponding 'isinstant' argument set to True, then
that variable is set to be instananeous (algebraic, not an ODE), i.e. s = sinf(v). The
taus function will then be ignored.

The resulting channel current will be of the form
    name.I = g * s^spow * s2^spow2 * (voltage - vrev)

Provide any additional Par or Fun objects necessary for the complete definition of the
channel kinetics in the optional parlist argument.

Returns a channel-type object by default, or some user-defined subclass if desired.
```

|  |  |
| --- | --- |
| makeChannel\_rates(name, voltage=`'``V``'`, s=None, isinstant=False, arate=None, brate=None, spow=1, s2=None, isinstant2=False, arate2=None, brate2=None, spow2=1, vrev=None, g=None, parlist=None, noauxs=True, subclass=<class 'PyDSTool.Toolbox.neuralcomp.channel'>, gamma1=None, gamma2=None) | source code |

```
Make an ionic membrane channel using the forward and backward rate formalism.

i.e., that the gating variable s has a differential equation in the form:
   s' = sa(v) * (1-s) - sb(v) * s
The channel may have up to two gating variables, each of which is given by an ODE.

If either gating variable has its corresponding 'isinstant' argument set to True, then
that variable is set to be instananeous (algebraic, not an ODE),
i.e. s = sinf(v) = a(v) / (a(v) + b(v))

The resulting channel current will be of the form
    name.I = g * s^spow * s2^spow2 * (voltage - vrev)

Provide any additional Par or Fun objects necessary for the complete definition of the
channel kinetics in the optional parlist argument.

Returns a channel-type object by default, or some user-defined subclass if desired.
```

|  |  |
| --- | --- |
| makeSoma(name, voltage=`'``V``'`, channelList=None, C=None, noauxs=True, subclass=<class 'PyDSTool.Toolbox.neuralcomp.soma'>, channelclass=<class 'PyDSTool.Toolbox.neuralcomp.channel'>) | source code |

Build a soma type of "compartment" from a list of channels
and a membrane capacitance C, using the local voltage name.

Specify a sub-class for the channel to target specific types to
summate, in case the user wants to have runtime control over which are
"active" in the dynamics (mainly related to the DSSRT
toolbox).

Returns a soma-type object by default, or some user-defined subclass
if desired.

|  |  |
| --- | --- |
| makeDendrite(name, voltage=`'``V``'`, channelList=None, C=None, noauxs=True, subclass=<class 'PyDSTool.Toolbox.neuralcomp.dendr\_compartment'>, channelclass=<class 'PyDSTool.Toolbox.neuralcomp.channel'>) | source code |

Build a dendrite type of "compartment" from a list of
channels and a membrane capacitance C, using the local voltage name.

Specify a sub-class for the channel to target specific types to
summate, in case the user wants to have runtime control over which are
"active" in the dynamics (mainly related to the DSSRT
toolbox).

Returns a dendrite-type object by default, or some user-defined
subclass if desired.

|  |  |
| --- | --- |
| makeNeurite(name, voltage=`'``V``'`, channelList=None, C=None, noauxs=True, subclass=<class 'PyDSTool.Toolbox.neuralcomp.dendr\_compartment'>, channelclass=<class 'PyDSTool.Toolbox.neuralcomp.channel'>) | source code |

Build a neurite type of "compartment" from a list of
channels and a membrane capacitance C, using the local voltage name.

Specify a sub-class for the channel to target specific types to
summate, in case the user wants to have runtime control over which are
"active" in the dynamics (mainly related to the DSSRT
toolbox).

Returns a neurite-type object by default, or some user-defined
subclass if desired.

|  |  |
| --- | --- |
| makePointNeuron(name, voltage=`'``V``'`, channelList=None, synapseList=None, C=None, noauxs=True) | source code |

Factory function for single compartment neurons ("point
neurons").

Returns a neuron type object from a list of ion channels and synapses
(if any are defined). The ion channels will internally be built into a
soma compartment type on the fly.

|  |  |
| --- | --- |
| makeNeuronNetwork(name, neuronList) | source code |

Factory function returning a network type object from a list of
compatible components (neurons). The neurons can be single or
multi-compartmental.

Currently untested!

  


|  |  |
| --- | --- |
| Variables Details | [hide private] |

### compatGens

Value:
:   ```
    ['Radau_ODEsystem',
     'ADMC_ODEsystem',
     'Vode_ODEsystem',
     'Euler_ODEsystem',
     'Dopri_ODEsystem']
    ```

### ScalarType

Value:
:   ```
    (<type 'int'>,
     <type 'float'>,
     <type 'complex'>,
     <type 'long'>,
     <type 'bool'>,
     <type 'str'>,
     <type 'unicode'>,
     <type 'buffer'>,
    ...
    ```

### cast

Value:
:   ```
    {<type 'numpy.int64'>: <function <lambda> at 0x10a8bb0>, <type 'numpy.
    int16'>: <function <lambda> at 0x10a8bf0>, <type 'numpy.complex128'>: 
    <function <lambda> at 0x10a8c30>, <type 'numpy.uint64'>: <function <la
    mbda> at 0x10a8c70>, <type 'numpy.complex256'>: <function <lambda> at 
    0x10a8cf0>, <type 'numpy.float32'>: <function <lambda> at 0x10a8d30>, 
    <type 'numpy.bool_'>: <function <lambda> at 0x10a8cb0>, <type 'numpy.u
    int8'>: <function <lambda> at 0x10a8db0>, <type 'numpy.int32'>: <funct
    ion <lambda> at 0x10a8eb0>, <type 'numpy.int8'>: <function <lambda> at
    ...
    ```

### index\_exp

Value:
:   ```
    <numpy.lib.index_tricks.IndexExpression object at 0x11543b0>
    ```

### nbytes

Value:
:   ```
    {<type 'numpy.int64'>: 8, <type 'numpy.int16'>: 2, <type 'numpy.comple
    x128'>: 16, <type 'numpy.uint64'>: 8, <type 'numpy.bool_'>: 1, <type '
    numpy.complex256'>: 32, <type 'numpy.float32'>: 4, <type 'numpy.int8'>
    : 1, <type 'numpy.uint8'>: 1, <type 'numpy.uint16'>: 2, <type 'numpy.o
    bject_'>: 4, <type 'numpy.float64'>: 8, <type 'numpy.int32'>: 4, <type
     'numpy.string_'>: 0, <type 'numpy.void'>: 0, <type 'numpy.float128'>:
     16, <type 'numpy.int32'>: 4, <type 'numpy.uint32'>: 4, <type 'numpy.u
    nicode_'>: 0, <type 'numpy.complex64'>: 8, <type 'numpy.uint32'>: 4}
    ```

### sctypeDict

Value:
:   ```
    {0: <type 'numpy.bool_'>,
     1: <type 'numpy.int8'>,
     2: <type 'numpy.uint8'>,
     3: <type 'numpy.int16'>,
     4: <type 'numpy.uint16'>,
     5: <type 'numpy.int32'>,
     6: <type 'numpy.uint32'>,
     7: <type 'numpy.int32'>,
    ...
    ```

### sctypeNA

Value:
:   ```
    {'?': 'Bool',
     'B': 'UInt8',
     'Bool': <type 'numpy.bool_'>,
     'Complex128': <type 'numpy.complex256'>,
     'Complex32': <type 'numpy.complex64'>,
     'Complex64': <type 'numpy.complex128'>,
     'D': 'Complex64',
     'F': 'Complex32',
    ...
    ```

### sctypes

Value:
:   ```
    {'complex': [<type 'numpy.complex64'>,
                 <type 'numpy.complex128'>,
                 <type 'numpy.complex256'>],
     'float': [<type 'numpy.float32'>,
               <type 'numpy.float64'>,
               <type 'numpy.float128'>],
     'int': [<type 'numpy.int8'>,
             <type 'numpy.int16'>,
    ...
    ```

### typeDict

Value:
:   ```
    {0: <type 'numpy.bool_'>,
     1: <type 'numpy.int8'>,
     2: <type 'numpy.uint8'>,
     3: <type 'numpy.int16'>,
     4: <type 'numpy.uint16'>,
     5: <type 'numpy.int32'>,
     6: <type 'numpy.uint32'>,
     7: <type 'numpy.int32'>,
    ...
    ```

### typeNA

Value:
:   ```
    {'?': 'Bool',
     'B': 'UInt8',
     'Bool': <type 'numpy.bool_'>,
     'Complex128': <type 'numpy.complex256'>,
     'Complex32': <type 'numpy.complex64'>,
     'Complex64': <type 'numpy.complex128'>,
     'D': 'Complex64',
     'F': 'Complex32',
    ...
    ```

### typecodes

Value:
:   ```
    {'All': '?bhilqpBHILQPfdgFDGSUVOMm',
     'AllFloat': 'fdgFDG',
     'AllInteger': 'bBhHiIlLqQpP',
     'Character': 'c',
     'Complex': 'FDG',
     'Datetime': 'Mm',
     'Float': 'fdg',
     'Integer': 'bhilqp',
    ...
    ```

  


| Home | Trees | Indices | Help | PyDSTool |
| --- | --- | --- | --- | --- |

|  |  |
| --- | --- |
| Generated by Epydoc 3.0.1 on Fri May 4 15:24:05 2012 | http://epydoc.sourceforge.net |
